# Chlorine gas inhalation manifesting with severe acute respiratory distress syndrome successfully treated by high-volume hemofiltration: A case report

**DOI:** 10.1097/MD.0000000000011708

**Published:** 2018-07-27

**Authors:** Lian Wang, Dingqian Wu, Jianqiang Wang

**Affiliations:** a Department of Thoracic Surgery; b Department of Emergency Medicine, The Second Affiliated Hospital, Zhejiang University School of Medicine; Research Institute of Emergency Medicine, Zhejiang University, Hangzhou; c Department of Respiratory Medicine, Jintan District Hospital of Traditional Chinese Medicine, Changzhou, China.

**Keywords:** acute respiratory distress syndrome, chlorine gas inhalation, high-volume hemofiltration

## Abstract

**Rationale:**

There have been occasional reports of respiratory dysfunction associated with acute chlorine gas inhalation. However, management of acute chlorine-related inhalation injury is largely empirical, supportive, and sometimes challenging.

**Patient concerns:**

A 43-year-old man was transferred to the emergency department because of accidental chlorine inhalation and rapidly progressive dyspnea.

**Diagnoses:**

The patient was diagnosed with acute respiratory distress syndrome due to chlorine gas exposure.

**Interventions:**

Because this patient had failed on conventional treatments including mechanical ventilation and high-dose intravenous corticosteroid therapy, we applied high-volume hemofiltration (HVHF).

**Outcomes:**

The patient recovered quickly after four sessions of HVHF and was discharged uneventfully on day 28.

**Lessons:**

HVHF is a potential method for improvement of chlorine-induced acute respiratory failure and worsening hypoxemia.

## 1 Introduction

There have been occasional reports of respiratory dysfunction associated with acute chlorine gas inhalation in both developed and developing countries.^[1]^ However, there is no specific antidote for chlorine and the mainstay of treatment is largely supportive.^[2]^ We report a case of chlorine gas inhalation manifesting severe acute respiratory distress syndrome (ARDS). Despite there are limited data to support the routine use of hemofiltration, the patient recovered quickly after 4 sessions of high-volume hemofiltration (HVHF) and was discharged uneventfully.

## 2 Case presentation

A 43-year-old man was transferred to the emergency department from local community hospital because of accidental chlorine inhalation and rapidly progressive dyspnea. Six hours before, a severe chlorine gas leak occurred at a metal recycling facility. The patient tried to control the site and thus stayed in the workshop for nearly 30 minutes without effective protection. He complained of tearing eyes, throat burning, nausea, and especially dyspnea in the initial hours. His symptoms were significantly worse than before though he was ventilated via a mask with 100% oxygen. He was a heavy smoker and had no history of cardiac disease.

**Figure 1:**
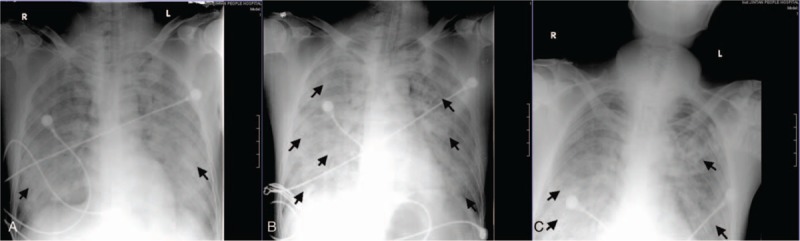
Serial CXRs taken at 1 (A), 2 (B), and 5 (C) days of admission. Arrow heads show interstitial and alveolar pulmonary edema. CXR = chest x-ray.

On examination he was mildly hypotensive, and had respiratory distress, and light yellowish, frothy nasal, and oral discharge. The arterial blood gas revealed: SaO_2_ 60%, PaO_2_ 36 mm Hg, PaCO_2_ 43 mm Hg, pH 7.25, BE −8 mmol/L. The chest x-ray (CXR) showed bilateral infiltrative opacities (Fig. 1A), which were interpreted as interstitial and alveolar pulmonary edema. ARDS due to chlorine gas exposure was diagnosed.^[1,3]^ He was promptly intubated and ventilated with a lung-protective strategy. Other treatments included infusion of dopamine to increase mean arterial blood, intravenous methylprednisolone 1000 mg and ulinastatin to inhibit pulmonary inflammatory response. However, there was no significant improvement in the overall clinical condition. High doses of vasoactive drugs were required to maintain blood pressure at 100/50 mm Hg. A repeat chest x-ray revealed worsening interstitial infiltrates (Fig. 1B). His HR decreased to 30 bpm when a tracheotomy was performed on day 3.

Subsequently, four sessions of high-volume hemofiltration (HVHF) at 65 mL/kg/h was started. Vascular access was obtained by cannulation of the right femoral vein using a double-lumen catheter (Hemo-Access, Gambro, Hechingen, Germany). Blood was pumped from the outflow lumen by the roller pump of a blood flow/air trap module (AK 10; Gambro, Lund, Sweden). It was delivered to a polyacrylonitrile AN69 filter (Filtral; Hospal, Germany) 1.60 m^2^ in membrane surface. The replacement fluids were administered at 6000 mL/h by a predilution method. The circuit was anticoagulated by fraxiparine (Sanofi Synthelabo, Hangzhou, China) at an initial 3000 U loading dose and a 400 U/h maintenance dose. The daily fluid balance was kept at a range of 500 to 1000 mL negative.

After the first session of HVHF, the PaO_2_ improved from 53 to 71 mm Hg and the PaO_2_/FiO_2_ ratio increased from 59 to 102 mm Hg. His PaO_2_ continued to increase to 148 mm Hg, with gradually reducing support levels of mechanical ventilation and doses of vasopressors in the following session. CXR showed resolving pulmonary infiltrates (Fig. 1C). During the fourth session of HVHF (day 6 after admission), a PaO_2_ of 174 mm Hg, which corresponded to a PaO_2_/FiO_2_ ratio of 316 mm Hg, was observed. His renal function reports before and after HVHF were normal. On day 11, the patient developed ventilator-associated pneumonia caused by multidrug-resistant acinetobacter baumannii and was treated with cefoperazone and sulbactam. Weaning from ventilation was achieved on day 17. He was discharged on day 21 and 28 from the intensive care unit (ICU) and hospital respectively.

The patient complained of manifested persistent cough that lasted for 4.5 months after discharge, and the pulmonary function tests revealed severe mixed ventilatory defect. He was managed with inhaled steroids and bronchodilatadors. The patients were asymptomatic and the pulmonary function was improved two years later.

## 3 Discussion

Acute exposure to chlorine gas due to accidental leaking is a well-recognized occupational health problem. In addition to the acidic environment resulting from chlorine interaction with the respiratory mucosa, the released free radicals and inflammatory mediators also destroy large airways as well as small airways and the alveolar-capillary structures.^[1]^ However, it is rare to develop ARDS and unlike in this case above, the requirement for mechanical ventilation is uncommon. Although there was no exact information about the inhaled concentration of chlorine gas in our case, such toxicity appears to occur at relatively longer duration and higher concentration of exposure, furthermore, a history of smoking seems to correlate to a more deleterious effect.^[4]^ Pulmonary edema was reported in 85% of the patients when the exposure level was 400 ppm.^[5]^

There is no specific antidote for chlorine and the mainstay of treatment is largely supportive.^[2]^ Current therapies are based on reported experience, such as supplemental humidified oxygen and assisted ventilation, β-adrenergic agents and bicarbonate inhalation, corticosteroid, intravenous frusemide, and aminophylline administration. However, no controlled trials have evaluated these therapies. Because our patient had failed on all previous treatments, we applied HVHF. The performance was impressive, showing that it is well tolerated, and potential for improvement of oxygenation and hemodynamics.

Extracorporeal blood purification is often indicated in the management of intoxications.^[6]^ Hemofiltration removes solutes by convection, which means that solutes from the plasma (both small and large molecules) were filtered through the membrane by the flow of water that has been induced by the pressure gradient. HVHF is defined as continuous high-volume treatment with an ultrafiltration rates more than 50 mL/kg/h a day or intermittent very high-volume treatment with an ultrafiltration rates of 100 to 120 mL/kg/h for a short period of 4 to 8 hours followed by conventional continuous venovenous hemofiltration (CVVH).^[7]^ There are many different hypotheses about how HVHF attenuate the excessive expression of systemic inflammatory mediators, restore immune homeostasis and finally improve clinical outcome.^[8]^ Although further large, randomized and controlled trials are needed, early promising studies have shown HVHF to improve hemodynamics and decrease vasopressor requirement in septic patients. Despite the frequent use of HVHF for acute kidney injury, much experimental and some clinical evidence suggest that HVHF effectively reduce the circulating levels of both pro- and anti-inflammatory mediators involved in systemic inflammatory response syndrome (SIRS) and multiorgan dysfunction syndrome (MODS), which is beneficial for critically ill patients with severe inflammatory states.^[7]^ Furthermore, the feasibility of a “negative balance” fluid management strategy during hemofiltration may help augment alveolar fluid clearance and relieve pulmonary edema.

To our knowledge, this is the first case using HVHF as salvage therapy in severe chlorine-induced ARDS and hypodynamic patient. Given the infrequency of respiratory failure caused by chlorine gas inhalation, and the lack of randomized controlled trials comparing different modalities of extracorporeal blood purification for treatment of poisoning, more evidence is needed to support its safe use in the future.

## 4 Conclusion

In conclusion, although the present prescription of HVHF are very practitioner-dependent and show great variability between institutions, this case demonstrates practical application of HVHF as a method to improve chlorine-induced ARDS and worsening hypoxemia.

## Acknowledgments

Thanks for this patient who consented to publish his case.

## Author contributions

**Conceptualization:** Jianqiang Wang.

**Funding acquisition:** Jianqiang Wang.

**Investigation:** Dingqian Wu, Jianqiang Wang.

**Writing – original draft:** Lian Wang.

**Writing – review & editing:** Jianqiang Wang.
